# Diagnosis of Congenital Cytomegalovirus (cCMV) in an Asymptomatic Neonate on Placental Pathology

**DOI:** 10.7759/cureus.30130

**Published:** 2022-10-10

**Authors:** Praveen Kumar Boddu, Pradeep Kumar Velumula, Suzanne M Jacques, Moumita S Choudhury, Bajaj Monika

**Affiliations:** 1 Children's Hospital of Michigan, Central Michigan University College of Medicine, Mt Pleasant, USA; 2 Pediatrics, Neonatal Perinatal Medicine, MercyOne Waterloo Medical Center, Waterloo, USA; 3 Pathology, Wayne State University Detroit Medical Center, Detroit, USA; 4 Neonatology, Central Michigan University College of Medicine, Mt Pleasant, USA

**Keywords:** term neonate, torch infections, placental pathology, cytomegalovirus (cmv), congenital cytomegalovirus

## Abstract

Congenital Cytomegalovirus (cCMV) is the most common intrauterine infection, with an incidence of 0.5% to 1.3% in the United States of America (USA). The majority of cCMV infections are asymptomatic at birth. In this case report, we present a full-term neonate who was admitted to the neonatal intensive care unit (NICU) for early onset sepsis and had an incidental finding in the placenta suggestive of Cytomegalovirus infection that was later confirmed on polymerase chain reaction (PCR) test in the blood. The infant was further evaluated for signs of CMV infection: complete blood count (CBC), head ultrasound, audiology, and ophthalmology exams were performed that did not show any abnormality. He was discharged home with audiology, ophthalmology, primary care, and infectious disease specialties follow-up appointments. Our case emphasizes the role of placental examination in looking for evidence of CMV infection so that infants can be diagnosed as well as followed up appropriately and necessary interventions can be provided on time for the best possible outcomes.

## Introduction

Cytomegalovirus (CMV) is a double-stranded deoxyribonucleic acid (DNA) virus in the Herpes virus family and is also known as human herpes virus 5 (HHV-5). CMV is a ubiquitous pathogen, with a 45% to 100% adult seroprevalence rate worldwide [1]. Prevalence among women of reproductive age is estimated to be 58% to 79% in North America and 86% globally [1]. Congenital Cytomegalovirus (cCMV) (cCMV) is the most common intrauterine infection seen, with an incidence of 0.5% to 1.3% in the USA [2]. It is also the most common cause of long-term pediatric disabilities compared to other causes such as fetal alcohol syndrome, Trisomy 21, neural tube defects, human immunodeficiency virus (HIV) and acquired immune deficiency syndrome (AIDS), Hemophilus influenzae b, and congenital Rubella syndrome [3]. There are no antenatal indicators to prognosticate the severity of CMV infection in neonates [4]. Although the majority of cCMV infections are asymptomatic at birth, these infants can develop progressive sensorineural hearing loss (SNHL) and other permanent sequelae later in life [5]. Currently, the diagnosis of CMV is made by targeted screening, where newborn babies are screened for CMV if they have any clinical features suggestive of CMV. Universal screening of CMV is not in practice [4]. While the primary diagnostic method is CMV DNA polymerase chain reaction (PCR) analysis in urine, blood, or saliva, we present a neonate who had no clinical features and had a placental diagnosis of CMV infection.

## Case presentation

A term male neonate was born at 39 weeks gestation to a 20-year-old Gravida 1 Para 1 mother. The mother had regular prenatal visits, and her antenatal ultrasound scans were all within normal limits. Her antenatal history was significant for positive Group B Streptococcus (GBS). She was diagnosed with chorioamnionitis due to a fever that started three hours before the delivery, foul-smelling amniotic fluid, maternal tachycardia, and leukocytosis. Her other lab work was negative, including HIV, hepatitis B, Gonococcus, and Chlamydia. The neonate was delivered via C-section because of non-reassuring fetal heart tones. He cried immediately after birth and had APGAR (appearance, pulse, grimace, activity, and respiration) scores of 6 and 9 at one and five minutes after birth, respectively. He received routine newborn care after delivery. His birth weight was 3635 grams (61%ile), length was 51 cm (52%ile), and head circumference was 35 cm (53%ile). His vital signs were within normal limits, and his physical examination showed no abnormalities. The neonate was admitted to the NICU for evaluation of early-onset sepsis in the setting of maternal chorioamnionitis. Sepsis screen and blood cultures were sent, and antibiotic therapy with intravenous Ampicillin and Gentamicin was started. Initial C-reactive protein (CRP) levels were elevated (16.6 mg/L) (Reference range <5 mg/L), and CBC showed a white blood cell count of 14.6K/cu.mm, hemoglobin of 16 mg/dL, and platelet count of 254K/cu.mm. The differential count was normal, and there was no left shift. The blood cultures were negative, and the follow-up CRP levels showed a decreasing trend. The neonate received seven days of antibiotic therapy, and he tolerated full enteral feeds.

**Figure 1:**
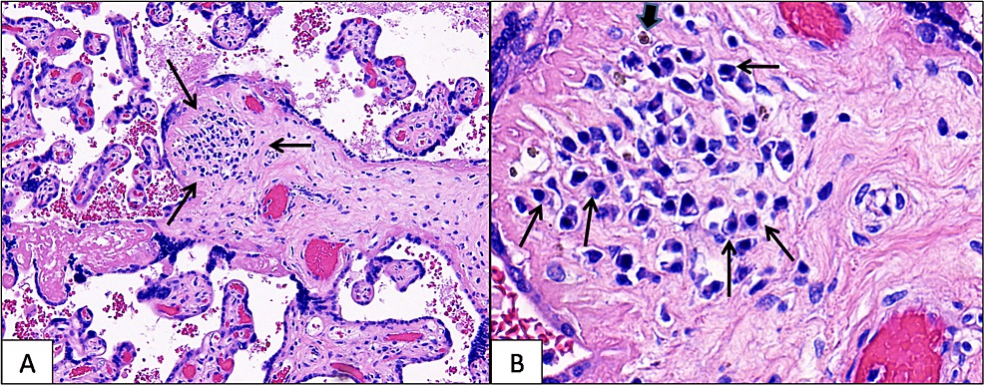
A: A cluster of plasma cells is present in a chorionic villus (arrows). The surrounding chorionic villi are unremarkable (H&E, magnification 100 x). B. Focal hemosiderin (short arrow) is seen adjacent to the plasma cells (long arrows) (H&E, magnification 400x) H&E: Hematoxylin and eosin stain

The placental pathology findings were as follows:

The trimmed placenta weighed 560 g, measured 15 x 16 x 2.5 cm, and was grossly unremarkable. Microscopically, rare chorionic villi in the mid-parenchyma showed lymphoplasmacytic chronic villitis (Figure 1A), and there was a rare, associated hemosiderin deposition (Figure 1B) in the affected chorionic villi. This pattern of inflammation is a strong histologic indicator of CMV infection. Therefore, a careful search for viral inclusions was done; however, no viral inclusions were identified. Immunohistochemical staining for CMV (done on two tissue blocks) was also negative. Additional placental findings included severe acute chorioamnionitis, focally necrotizing (acute maternal inflammatory response stage 3, grade 2) and acute funisitis (arteritis and phlebitis), and severe acute chorionic vasculitis (acute fetal inflammatory response stage 2, grade 2), findings consistent with ascending bacterial infection.

The neonate was evaluated for confirmation of a cCMV infection and other possible complications associated with it. The blood CMV PCR was positive, and his liver function tests were within normal reference ranges: total bilirubin 1.14 mg/dL, direct bilirubin 0.23mg/dL, aspartate aminotransferase (AST) 45 units/L, alanine transaminase (ALT) 14 units/L, alkaline phosphatase (ALP) 187 units/L. The head ultrasound noted lenticulostriate mineralizing vasculopathy, and the brain MRI was reported as normal for his age. On ophthalmological examination, there were no features suggestive of CMV chorioretinitis. On automated auditory brainstem response (AABR) evaluation, performed on the seventh day after birth, the neonate passed his hearing screen in bilateral ears. He was discharged home on day eight after birth with follow-up appointments scheduled with primary care, audiology, ophthalmology, and pediatric infectious disease sub-specialists.

## Discussion

The infant discussed in the case report was a full-term infant with no features suggestive of cCMV infection and was diagnosed based on placental pathology findings. On further evaluation, no other organ systems were involved, and he was discharged home with appropriate follow-up. The intrauterine transmission of CMV can happen with either maternal primary or nonprimary infection. Nonprimary maternal infection is usually due to the reactivation of CMV or reinfection with a different strain of the CMV. The risk of congenital transmission and the severity of the neonatal infection are substantially high with maternal primary infection. Although the risk of transmission is high with a primary CMV infection, most congenital CMV infections result from a nonprimary CMV infection because of the high prevalence of seropositivity in the general population [2]. Maternal CMV infection during the first half of pregnancy is associated with a lower risk of transmission but higher rates of long-term sequelae [4]. The patient's mother in the discussion was never diagnosed with CMV in the past, and her pregnancy was uneventful.

CMV infection can be transmitted to the fetus either across syncytiotrophoblasts leading to infection of the underlying cytotrophoblasts and/or via invasive cytotrophoblasts within the uterine wall [5]. In a study of 35 women whose babies were diagnosed with cCMV infection, it was found that compared to asymptomatic cCMV infections, a more significant number of symptomatic cCMV infections were associated with chronic villitis [6]. Changes in the villi, including lymphoplasmacytic villitis, avascular villi, and fibrotic villi, are described in the placental microscopy of cytomegalovirus infection [6]. CMV-infected placentas typically show lymphoplasmacytic villitis, and additional characteristic findings include hemosiderin deposits, fibrosis, and calcification [7,8]. Viral inclusions can sometimes be identified in the nucleus and cytoplasm of placental endothelial and stromal cells, and the Cowdry-A intranuclear inclusions (owl-eye cells) allow a definite diagnosis of CMV infection [8]. Even in the absence of viral inclusions, the finding of plasma cells and hemosiderin in chorionic villi is a strong predictor of CMV infection, as this current case illustrates. The morphologic features of infected placentas have been shown to change according to gestational age. Viral inclusions are more likely to be abundant in the second trimester but are scarce in the third trimester [8].

The infant in the discussion had no symptoms of cCMV at birth. Only 10% of cCMV infections are known to be symptomatic at birth [9]. The symptoms of cCMV can range from intrauterine growth retardation (IUGR), central nervous system abnormalities like microcephaly, cortical malformations, ventriculomegaly, periventricular calcifications, SNHL, chorioretinitis, hepatosplenomegaly, transaminitis, direct-hyperbilirubinemia, and petechiae/thrombocytopenia. Isolated SNHL is classified as a subgroup of asymptomatic infection [10]. Infants with SNHL need hearing screening at frequent intervals as SNHL is the most common sequelae of cCMV and 40% of SNHLs are late onset, i.e., after one month of age. The Incidence of SNHL is 25% to 65 % in symptomatic and 6% to 25% in asymptomatic infants [11]. Other permanent sequelae, including microcephaly, motor deficits, and chorioretinitis, have also been reported in infants with asymptomatic cCMV [12].

 It is recommended that all infants with cCMV should undergo blood counts, bilirubin and transaminase levels, renal function tests, brain imaging (ultrasound, computed tomography (CT), or magnetic resonance imaging (MRI)), and complete diagnostic audiology assessment [4]. As the head ultrasound of our patient noted lenticulostriate vasculopathy, an MRI of the brain was obtained for further evaluation that did not show any abnormality.

There are specific indications for the treatment of cCMV. Valganciclovir is the preferred drug for cCMV [13]. Our patient had an asymptomatic cCMV infection and did not meet the criteria for valganciclovir therapy. For such patients, hearing testing is recommended at regular intervals at 4, 6, 9, 12, 15, 18, 24, and 30 months, in addition to the routine screening done at 4, 5, 6, 8, and 10 years of age [14]. There are no predictors that have been described to identify patients with delayed onset or progressive SNHL [4].

## Conclusions

Although the screening for cCMV is done by CMV DNA PCR analysis, placental pathology plays an adjunctive role in diagnosing cCMV infection. Placental microscopic examination can identify neonates with asymptomatic CMV infection, prompting further evaluation and adequate follow-up. As asymptomatic cCMV infection increases the risk for progressive hearing loss, timely diagnosis can help with regular follow-up and early detection.
